# Probiotics in the management of radiation-induced oral mucositis

**DOI:** 10.3389/fcimb.2024.1477143

**Published:** 2024-09-18

**Authors:** Yixuan Li, Zixia Li, Shuhao Zheng, Xin Xu

**Affiliations:** State Key Laboratory of Oral Diseases & National Center for Stomatology & National Clinical Research Center for Oral Diseases & Department of Cariology and Endodontics, West China Hospital of Stomatology, Sichuan University, Chengdu, China

**Keywords:** radiation-induced oral mucositis, probiotics, oral microbiota, microbial dysbiosis, microbial ecology, head and neck cancer

## Abstract

Oral mucositis is a common and debilitating oral complication in head and neck cancer patients undergoing radiotherapy, resulting in diminished quality of life and potential treatment disruptions. Oral microbiota has long been recognized as a contributing factor in the initiation and progression of radiation-induced oral mucositis (RIOM). Numerous studies have indicated that the radiation-induced oral microbial dysbiosis promotes the occurrence and severity of oral mucositis. Therefore, approaches that modulate oral microbial ecology are promising for the management of RIOM. Probiotics as a relatively predicable and safe measure that modulates microecology have garnered significant interest. In this review, we discussed the correlation between RIOM and oral microbiota, with a particular focus on the efficacy of probiotics in the control of RIOM, in order to provide novel paradigm for the management of this disease.

## 1 Introduction

Radiation-induced oral mucositis (RIOM) refers to an affliction affecting the mucosal epithelium within the oral cavity, pharynx, and larynx resulting from the implementation of radiotherapy. RIOM is a common complication that occurs during and shortly after radiotherapy for patients, affecting nearly all patients with head and neck cancer (Maria et al., 2017; Berger et al., 2018). RIOM is primarily characterized by symptoms such as congestion, erythema, ulceration, erosion, and fibrosis of the oral mucosa. These manifestations are often accompanied by intense pain, difficulty in swallowing, altered taste perception, and potential secondary infections. Such symptoms can modify the nutritional uptake of individuals and reduce their overall quality of life, potentially leading to disruptions in cancer treatment (Anderson et al., 2021). The Multinational Association of Supportive Care in Cancer and International Society of Oral Oncology (MASCC/ISOO) recommend clinical strategies for the management of RIOM, including basic oral care, non-steroidal anti-inflammatory drugs, mucosal protective agents, growth factors and cytokines, antimicrobials, painkillers, and others (Elad et al., 2022). Among these, palifermin (keratinocyte growth factor-1) is the only drug that is approved by the FDA to relieve chemotherapy-induced oral mucositis in patients with malignant hematological diseases. However, there remains a significant need for safe and effective means to prevent and treat RIOM.

The human oral cavity, as one of the five major microbial reservoirs in the human body, hosts up to 700 species of bacteria (Kilian et al., 2016). Accumulating evidence has shown that oral microbiota undergoes dynamic changes during radiotherapy, shifting from predominantly oral *Streptococci* to a more pathogenic gram-negative flora that releases endotoxins. Particularly, the increase in gram-negative bacteria has been shown to exacerbate the severity of RIOM in patients with nasopharyngeal carcinoma (Zhu et al., 2017; Hou et al., 2018). Our recent animal study has also shown that oral cavity of RIOM mice harbors a dysbiotic microbiota characterized by the overgrowth of oral anaerobes (Wang et al., 2021). Attempts to use antimicrobials to eliminate oral flora and thus prevent and control RIOM have been proposed, but with limited success, and long-term use of antimicrobials may further aggravate microbial dysbiosis (Wijers et al., 2001; Stokman et al., 2003). It is widely believed that a stable and diverse microbiota is essential for the physiological processes and mucosal immune function of the host (Honda and Littman, 2016; Vasconcelos et al., 2016). Hence, measures that promote or restore oral microecology are promising for the clinical management of RIOM. Probiotics that can modulate microecology and possess anti-inflammatory and immunomodulatory activities have shown positive effects on the prevention and treatment of radiotherapy and/or chemotherapy-induced mucositis in both oral cavity and gastrointestinal (GI) tract (Azad et al., 2018). Recent studies from our group and others have also demonstrated that probiotics, either delivered *per oral* or topically, can significantly reduce the incidence, duration, severity and time to onset of RIOM with acceptable safety (Sharma et al., 2012; Jiang et al., 2019; Xia et al., 2021; Manifar et al., 2022; Mirza et al., 2022; Peng et al., 2024). In this paper, we critically review the role of oral microbial ecology in the development of RIOM, introduce the recent advance in the application of probiotics to the control of this disease, and discuss the current limitations and future efforts to promote the clinical translation of probiotics in the management of RIOM.

## 2 The role of oral microbiota in RIOM

According to Sonis, the pathophysiology of RIOM is a dynamic process consisting of five consecutive overlapping phases: initiation, primary damage response (inflammatory upregulation and activation), amplification of the damage responses, ulceration, and healing (Sonis, 2004; Elad et al., 2022). Radiotherapy can directly injure DNA and lead to apoptosis of epithelial cells, while oxidative stress generates reactive oxygen species (ROS) that further activate pathways such as the nuclear factor-κB (NF-κB) pathway associated with mucositis, leading to excessive production of pro-inflammatory cytokines and further damage to basal epithelial cells and submucosal tissues. Subsequently, bacterial, viral, and fungal colonization is promoted, further exacerbating tissue damage and superimposing secondary infections that aggravate mucosal lesions. The interplays between the oral microbiota and damaged mucosal tissues play an important role in the development of RIOM.

Dynamic alterations in the oral microbiota during radiotherapy have been reported for years (Zhu et al., 2017; Hou et al., 2018; Reyes-Gibby et al., 2020; Vesty et al., 2020; Mojdami et al., 2022). Uzel et al. used a culture-based approach to quantify the dynamic change of bacterial load in a hamster model of RIOM, and they found that bacterial counts increased but lagged behind RIOM development (Sonis, 2009). Consistently, Musha et al. found the bacterial counts in the saliva gradually increased in head and neck cancer patients undergoing radiotherapy, and that patients with bacterial counts exceeded the mean before radiotherapy tended to develop faster onset and slower healing (Musha et al., 2022).

In addition to total bacterial count, compositional change in oral microbiota may be more related to RIOM. Using 16S rRNA sequencing, Zhao et al. found that the number of species in the oral microbiota of mice with severe RIOM was reduced, and the α-diversity index was significantly reduced (Zhao et al., 2023). Another study reported that the β-diversity of oral microbiota was significantly altered between pre-radiotherapy and mid-radiotherapy in patients with head and neck cancer (Mojdami et al., 2022). More importantly, several studies have reported that the detection rate and abundance of specific opportunistic bacteria were increased in RIOM patients, including *Enterococci*, *Escherichia coli*, *Staphylococcus aureus*, *Staphylococcus epidermidis*, *Pseudomonas aeruginosa*, *Klebsiella pneumoniae (* Gaetti-Jardim et al., 2011; Panghal et al., 2012; Sonalika et al., 2012; Almståhl et al., 2018; Subramaniam and Muthukrishnan, 2019). Almståhl et al. reported that bacteria associated with oral health like *Streptococci* and *Neisseria* were reduced, while microorganisms associated with mucosal infections like *Enterococcus* and *Candida* were increased on the tongue and buccal mucosa of patients undergoing radiotherapy, and the alteration of microbiota may potentially trigger the development of RIOM (Almståhl et al., 2018). Vesty et al. identified a positive correlation of the presence of ≥ grade 2 oral mucositis with an increase of specific species including *Capnocytophaga leadbetteri*, *Neisseria mucosa*, *Olsenella uli, Parviomonas micra* and *Tannerella forsythia* in the saliva of patients at the early stages of radiotherapy (Vesty et al., 2020). Additionally, Hou et al. reported that the abundance of *Prevotella*, *Fusobacterium*, *Treponema* and *Porphyromonas* showed markedly synchronized dynamic changes, with peaks frequently coinciding with the onset of severe oral mucositis (Hou et al., 2018).

Consistently, radiotherapy also induces functional change of the oral microbiota. Subramaniam et al. reported upregulation of antibiotic-resistant genes in isolated bacterial colonies from patients receiving radiotherapy, including MCR-1 (mobilized colistin resistance), VIM2 (β-lactam resistance), TET(K) (tetracycline resistance) and bla(KPC) (carbapenem resistance) (Subramaniam and Muthukrishnan, 2019). The spread of antibiotic-resistant genes between bacteria may further increase the risk of complicated infections and potentially cause the failure of conventional treatments (Zhang et al., 2022). Furthermore, an *in vitro* study found that γ-irradiation altered the functionality of resident oral microorganisms by inducing biofilm formation and increasing bacterial virulence, which can be a risk factor for the development of RIOM (Vanhoecke et al., 2016).

Although there have been many studies indicated the correlation of microbial dysbiosis and RIOM, the causal relationship between oral microbiota and RIOM has yet to be fully evidenced. Oral microbial transplantation (OMT) is a promising method to demonstrate the potential causal effect of microbiota on diseases (Xiao et al., 2021; Li et al., 2022). Xiao et al. transplanted oral microbiota obtained from healthy mouse donors to the mice exposed to localized head and neck radiotherapy, and they found that OMT ameliorated RIOM in mice by countering the radiation-induced microbial alterations as well as inflammation in tongue and plasma (Xiao et al., 2021). In addition, sterile rats induced by antibiotics showed reduced tongue ulcer area and shorter duration of severe oral mucositis after receiving nasal radiotherapy (Al-Qadami et al., 2022), further support the causal effect of oral microbiota in the development of RIOM.

Therefore, it can be speculated that radiotherapy *per se* induces an early mucosal inflammatory response along with alterations in oral microbiota, and the dysbiotic microbiota in turn amplifies inflammatory response induced by radiotherapy, and ultimately promotes the development of RIOM.

## 3 Probiotics in the management of RIOM

Management of RIOM includes a synthesis of prophylaxis, symptom control, supportive care, and emerging therapies. Current strategies primarily focus on using low-dose radiation techniques, low-power laser therapy, and oral care as preventive measures, while symptom relief and complication reduction are achieved through oral moisturizers, analgesics, nutritional support, and infection control (Lalla et al., 2014; Hong et al., 2019; Elad et al., 2020; Grant et al., 2020; Wu and Cheng, 2022). In addition, a number of emerging therapeutic approaches such as growth factors, natural products (e.g. honey), and immunomodulators are under investigation to further improve the management of RIOM (Lalla et al., 2014; Yang et al., 2019; Elad et al., 2020; Wu and Cheng, 2022). These strategies benefit the prevention and symptom alleviation, but have limited efficacy and present challenges such as potential side effects and high costs. Therefore, there is still a need to explore more effective, relatively safe and economical strategies to promote the management of RIOM.

**Figure 1:**
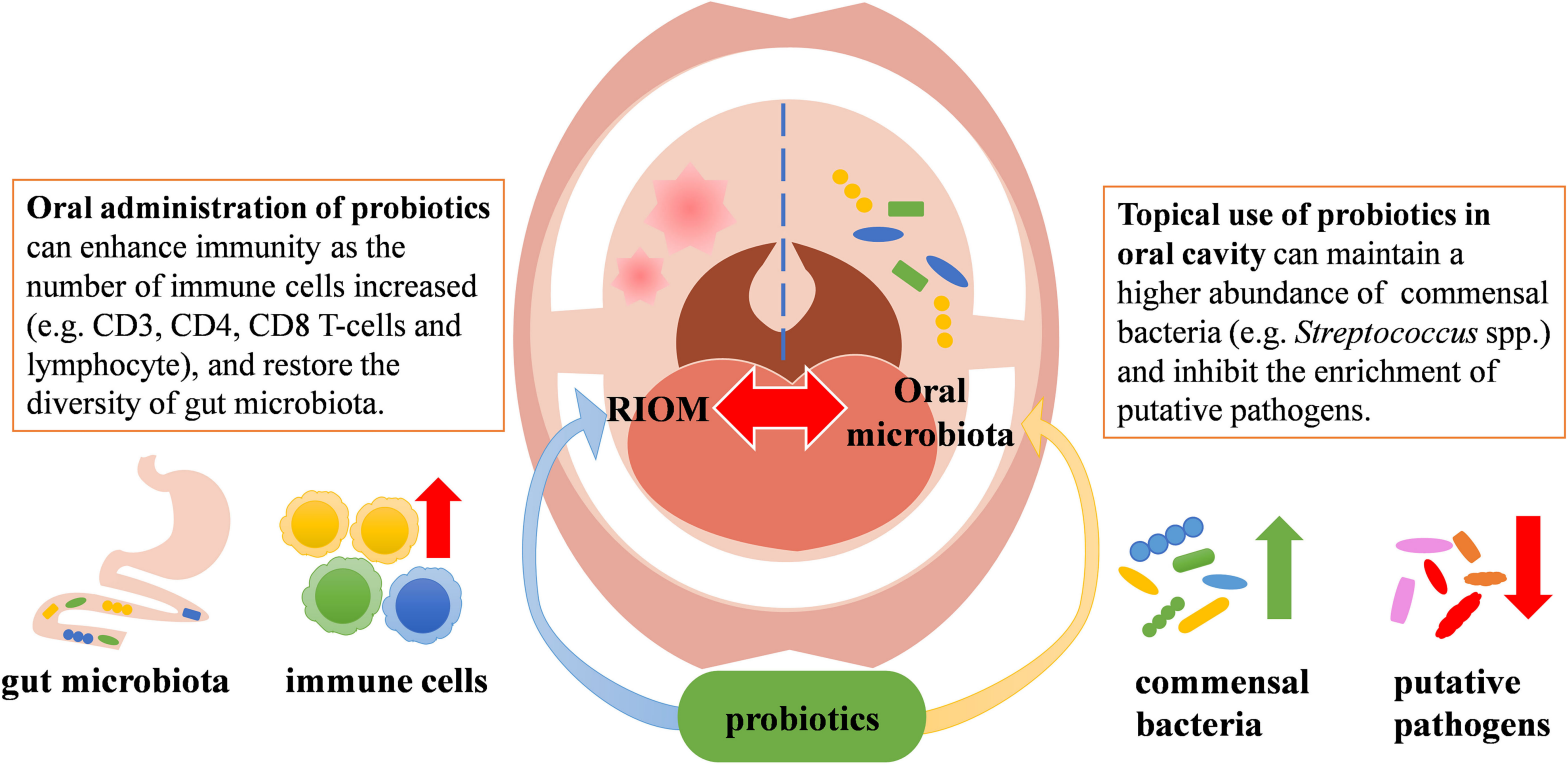
Potential mechanisms of probiotics in the management of RIOM.

Probiotics are a group of active microorganisms that can promote the health of hosts when administered in appropriate approach (Suez et al., 2019). Studies have shown that probiotics can improve microecological balance and have anti-inflammatory and immunomodulatory effects (Azad et al., 2018). They may exert beneficial effects by producing antimicrobial substances, competing with pathogens for adhesion and nutrition, participating in host immunomodulation, and inhibiting the production of bacterial toxins. In addition, probiotics can inhibit apoptosis of epithelial cells by agonizing toll-like receptors (TLRs) (Riehl et al., 2019). It has also been shown that probiotics can initiate T and B cell memory, trigger adaptive immunity, and activate immune system, which in turn stimulates the production of salivary glycoproteins and antimicrobial peptides, and thus protect the oral mucosa from damage (Thomas et al., 2017). To date, several probiotics have shown beneficial effects against RIOM. Although the mechanisms by which probiotics benefit RIOM have not been fully elucidated, data from numerous animal studies and clinical trials have demonstrated that probiotics have positive effect on the management of RIOM likely via modulating microbiota or regulating immune response (**Figure 1**). Currently available clinical trials using probiotics to treat RIOM are shown in **Table 1**.

**Table 1:** Clinical trials on probiotics for the management of RIOM.

Type of probiotic	Source	possible mechanism	Study Population (N)	Reference
*Lactobacillus brevis* CD2	gut-derived	To modulate gut microbiota	Patients with head and neck squamous cell carcinoma (N=200)	(Sharma et al., 2012)
		_	Patients with head and neck carcinoma (N=75)	(De Sanctis et al., 2019)
Mixed probiotics (consisted of *Bifidobacterium longum, Lactobacillus lactis, and Enterococcus faecium)*	gut-derived	To maintain bacterial homeostasis of gut and modulate human immune response.	Patients with nasopharyngeal carcinoma (N=99)	(Jiang et al., 2019)
Mixed probiotics (consisted of *L. plantarum* MH-301, *B. animalis* subsp. *Lactis* LPL-RH, *L. rhamnosus* LGG-18, and *L. acidophilus*)	gut-derived	To improve the immunity of patients and regulate gut microbiota.	Patients with nasopharyngeal carcinoma (N=85)	(Xia et al., 2021)
*Bacillus clausii* UBBC07	gut-derived	To rescue microbial dysbiosis, exert anti-inflammatory effects, and promote host immunity.	Patients with head and neck cancer (N=46)	(Mirza et al., 2022)
Synbiotic mouthwash (consisted of *Bifidobacterium breve*, *Bifidobacterium longum*, *Lactobacillus acidophilus*, *Lactobacillus casei*, *Lactobacillus bulgaricus*, *Lactobacillus rhamnosus*, *Streptococcus salivarius* subsp. *thermophiles*, and fructooligosaccharide as a prebiotic)	gut-derived	To regulate oral microbiota and enhance local immune response.	Patients with oral squamous cell carcinoma (N=64)	(Manifar et al., 2022)
*Streptococcus salivaris* K12	oral cavity-derived	To maintain the abundance of oral commensals during radiotherapy and inhibit the growth of opportunistic pathogens.	Patients with head and neck malignant tumor (N=160)	(Peng et al., 2024)

### 3.1 Effect of gut-derived probiotics on RIOM

A randomized, double-blind, placebo-controlled study by Sharma et al. included 200 patients with head and neck squamous carcinoma undergoing radiotherapy and concurrent chemotherapy. The study found that daily intake of lozenges containing *Lactobacillus brevis* CD2 during radiotherapy reduced the incidence of grade 3 and 4 RIOM (severe RIOM) (Sharma et al., 2012). However, a multicenter prospective randomized study by De Sanctis et al. failed to demonstrate the protective effects of *Lactobacillus brevis* CD2 against RIOM in patients receiving intensity-modulated radiotherapy and concurrent chemotherapy (De Sanctis et al., 2019). The authors speculated that this divergent result from Sharma’s data may be attributed to the smaller sample size, the difference in radiotherapy modality, and the use of sodium bicarbonate mouthwash instead of placebo in the control group (De Sanctis et al., 2019). A randomized double-blind placebo-controlled trial by Jiang et al. administered mixed probiotics (capsules containing *Bifidobacterium longum*, *Lactobacillus lactis*, and *Enterococcus faecium*) to patients with nasopharyngeal carcinoma treated with concurrent radiotherapy and chemotherapy. They found that the administration of the probiotics resulted in a decrease in the incidence and severity of oral mucositis, an increase in the CD3, CD4, CD8 T-cells and lymphocyte levels. Meanwhile, supplementation with the mixed probiotics promoted the restoration of intestinal microbial diversity, thereby improving the efficacy and reducing the mucosal toxicity of radiotherapy and chemotherapy (Jiang et al., 2019). Another clinical study along with a rat study by Xia et al. also supported the protective effect of mixed probiotics (capsules containing *L. plantarum* MH-301, *B. animalis* subsp. *Lactis* LPL-RH, *L. rhamnosus* LGG-18, and *L. acidophilus*) on oral mucositis. Their data showed that application of mixed probiotics led to an improved immunity (increased CD3, CD4, and CD8 T-cells) in patients with nasopharyngeal carcinoma receiving radiotherapy and improved intestinal homeostasis in head and neck irradiated patients and rats (Xia et al., 2021). Mirza MA et al. conducted a randomized, double-blind, placebo-controlled study with 46 head and neck cancer patients undergoing radiotherapy who were given *Bacillus clausii* UBBC07 (in the form of an oral suspension of 2 billion spores) twice daily. They found that *Bacillus clausii* UBBC07 delayed the onset of RIOM, reduced the duration of RIOM remission, and prevented severe oral mucositis via restoring microbial equilibrium and exerting anti-inflammatory and immune modulatory activity (Mirza et al., 2022). Of note, probiotics in the aforementioned studies were primarily administered *per oral*, which more likely act on the GI tract via enhancing host immunity and restoring gut homeostasis, and thus indirectly promote the healing of RIOM. A recent clinical trial by Marnifar et al. reported that a synbiotic mouthwash, which contained *Bifidobacterium breve*, *Bifidobacterium longum*, *Lactobacillus acidophilus*, *Lactobacillus casei*, *Lactobacillus bulgaricus*, *Lactobacillus rhamnosus*, *Streptococcus salivarius* subsp. *thermophiles* and fructooligosaccharide (function as a prebiotic), significantly reduced the occurrence and severity of RIOM (Manifar et al., 2022). Although the impact on oral microbial ecology was not reported, the protective effects of this synbiotic mouthwash against RIOM are likely accredited to the direct action on oral microbiota and local immune response in the oral cavity.

### 3.2 Effects of oral cavity-derived probiotics on RIOM

As oral cavity is relatively a conserved ecological niche that may be exclusive to foreign colonizers, a reliable and persistent colonization of gut-derived probiotics administered *per oral* is arguable (Yli-Knuuttila et al., 2006; Meurman and Stamatova, 2007; Caglar et al., 2009). Hence, probiotic strains isolated from oral cavity may have an innate advantage of reliable/persistent colonization in the oral cavity and thus yield a predictable long-term protective effect against oral mucositis.

*Streptococcus salivarius* K12, a commensal strain isolated from the oral cavity of infants that produces two bacteriocins, i.e. salivaricin A2 and salivaricin B (Tagg, 2004). *S. salivarius* K12 has been used in the clinical treatment of oral candidiasis, tonsillitis, pharyngitis, halitosis and otitis media in infants and young children accredited to its potent oral colonizing capability, low pathogenicity, and superior ecological modulating and immune-modulating activity (Burton et al., 2006; Cosseau et al., 2008; Burton et al., 2011; Burton et al., 2013; Zupancic et al., 2017; Yoo et al., 2020). Data from our previous animal study have shown that topical application of *S. salivarius* K12 to the oral cavity of radiation-induced mice significantly alleviated RIOM via inhibiting the overgrowth of oral anaerobes (Wang et al., 2021). Our recent prospective randomized controlled clinical trial recruited 160 head and neck cancer patients undergoing intensity-modulated radiotherapy alone or concurrent chemotherapy, and we demonstrated that topical use of *S. salivarius* K12 lozenge effectively reduced the incidence of oral mucositis, delayed its onset, shortened its duration, and alleviated the severity of RIOM. More importantly, topical use of *S. salivarius* K12 maintained a higher abundance of *Streptococcus* spp. and inhibited the enrichment of putative pathogens during radiotherapy (Peng et al., 2024). Of note, in this study, we also observed that approximately 1/3 of the *S. salivarius* K12-treated patients still developed severe oral mucositis. This heterogeneity in treatment outcome may be due to the difference in the treatment-naïve microbiota that has varied resistance to radiation and probiotic interventions. In addition, as *S. salivarius* K12 not only exerts probiotic activity via modulating microecology but also benefits the host through anti-inflammatory and immunomodulatory capability, the heterogeneity in host response to the pleiotropic effects of *S. salivarius* K12 may also explain the varied treatment outcome by this oral probiotic strain.

## 4 Future perspectives

Despite the current available evidence that support the beneficial effects of probiotics on RIOM, there still exist several issues to be addressed with future efforts. Firstly, radiotherapy can cause a wide range of concurrent oral complications other than RIOM, such as taste dysfunction, rampant caries and xerostomia, etc. Taste dysfunction occurs in 70-90% of patients during radiotherapy to the head and neck region, though it may recover partially after radiotherapy but for some cases it could last for months to years (Vissink et al., 2003; Gunn et al., 2021). Radiation-related caries is a typical example of rampant caries that is usually observed in patients after radiotherapy with a rapid onset and widespread involvement (Vissink et al., 2003). Meanwhile, the incidence of radiation-induced salivary gland injury after conventional radiotherapy for nasopharyngeal carcinoma is nearly 100%, leading to dysphagia and xerostomia that further aggravate radiation-related caries (Epstein et al., 2012; Mercadante et al., 2017). In addition to these radiation-related oral complications, the risk of osteoradionecrosis is also challenging for the operational procedures such as tooth extraction on patients undergoing radiotherapy (Vissink et al., 2003; Epstein et al., 2012). Although certain strains of *S. salivarius*, *L. rhamnosus* and *L. plantarum* have also shown anti-caries and anti-infection potentials in animal models or clinical trials (Matsubara et al., 2016; Seminario-Amez et al., 2017; Bustamante et al., 2020; D’Agostino et al., 2024), whether application of these probiotics can effectively tackle these concurrent oral complications and clinical challenges other than RIOM has yet to be investigated in well-controlled clinical trials.

Secondly, the underlying mechanisms by which probiotics ameliorate RIOM remain inadequately elucidated. Future efforts to elucidate the mechanisms on molecular and cellular levels through which probiotics exert their effects are in urgent need for the better clinical translation. In the treatment of radiation-induced mucositis in GI tract, *L. rhamnosus* GG has been identified to release lipoteichoic acid (LTA), which binds to TLR2 and actives macrophages to produce CXCL12. CXCL12 then binds to CXCR4, triggering the migration of COX-2 expressing mesenchymal stem cells (MSCs) to the lamina propria adjacent to the crypt epithelial cells, thereby protecting the intestinal epithelial stem cells from radiation damage by releasing PGE_2_ (Ciorba et al., 2012; Riehl et al., 2019). Consistently, *L. plantarum* can promote DNA damage repair in crypt cells via activating the farnesoid X receptor-fibroblast growth factor 15 (FXR-FGF15) signaling, thus reducing radiation-induced intestinal damage (Jian et al., 2022). Whether probiotics exert radioprotective effects on oral mucosa via similar mechanisms or other oral mucosa-specific mechanisms exist need further elucidation. In addition, *L. brevis* CD2 can produce high levels of arginine deiminase and sphingomyelinase (Di Marzio et al., 2001). The former can reduce the availability of arginine in the oral cavity and lead to a reduction in nitric oxide, resulting in lower levels of inflammation (Riccia et al., 2007). Sphingomyelinase is able to hydrolyze platelet activating factor that acts as an inflammatory cytokine associated with RIOM (McManus et al., 1993; Duan, 2006). Of note, there still lacks in-depth mechanistic studies with respect to the protective effects of probiotic on RIOM, future studies with proper animal models and genetic tools are still needed to further delineate the radioprotective effects of probiotics at molecular and cellular levels.

Besides, inconsistent data have been noted in literature regarding the efficacy of probiotics on RIOM. For example, the incidence of severe oral mucositis in the probiotics-treated group varied widely across the literature (25%~54.2%) (Sharma et al., 2012; De Sanctis et al., 2019; Xia et al., 2021; Mirza et al., 2022; Peng et al., 2024). In addition, the onset and duration of mucositis after treatment also varied across studies (De Sanctis et al., 2019; Mirza et al., 2022; Peng et al., 2024). These discrepancies may be attributed to the wide variation of sample size in different trials (ranging from 46 to 200 patients) (Sharma et al., 2012; De Sanctis et al., 2019; Jiang et al., 2019; Xia et al., 2021; Manifar et al., 2022; Mirza et al., 2022; Peng et al., 2024). In addition, variations in the regimens of radiotherapy may also account for this discrepancy. For example, Sharma’s study used a 2D radiation therapy technique, whereas the other trials used intensity-modulated radiation therapy (IMRT) which reduced the dose of radiation for better protection of the oral cavity and thus may significantly affect the onset time, duration and severity of oral mucositis (Sharma et al., 2012; De Sanctis et al., 2019; Jiang et al., 2019; Xia et al., 2021; Manifar et al., 2022; Mirza et al., 2022; Peng et al., 2024). The types of cancer in the subjects recruited in different studies may also have impact on the treatment outcome reported in literature. Jiang and Xia’s study included only patients with nasopharyngeal cancer, Marnifar’s study included only patients with oral squamous cell carcinoma, while other studies included multiple types of head and neck cancer (Sharma et al., 2012; De Sanctis et al., 2019; Jiang et al., 2019; Xia et al., 2021; Manifar et al., 2022; Mirza et al., 2022; Peng et al., 2024). Besides, the systemic health of patients, the frequency/dosage/duration and delivery mode of probiotics could also significantly confound the efficacy of probiotics against RIOM, which warrant more controlled clinical trials in the future to generate high quality clinical evidence.

In addition, although no death or serious adverse reactions related to probiotics supplementation have been documented in the currently available clinical trials (Sharma et al., 2012; De Sanctis et al., 2019; Jiang et al., 2019; Xia et al., 2021; Manifar et al., 2022; Mirza et al., 2022; Peng et al., 2024), long-term follow-up is still needed to comprehensively assess its safety and efficacy. Potential side effects of probiotics include gastrointestinal reactions and flu-like symptoms (Snydman, 2008; Doron and Snydman, 2015). Probiotics may also translocate to cause infections in the recipient, and in the worst cases may even cause fatal sepsis (Snydman, 2008; Doron and Snydman, 2015; Zawistowska-Rojek and Tyski, 2018). In addition, as probiotics can modulate the body’s immune response, it has the potential to stimulate overactions of immune system and cause fever or arthritis (Zawistowska-Rojek and Tyski, 2018). The transfer of resistance genes between probiotics and other commensal or pathogenic bacteria in the body is another potential risk of probiotic use (Snydman, 2008; Zawistowska-Rojek and Tyski, 2018). Therefore, cautions should still be taken particularly when probiotics are applied to immunocompromised or critically ill patients.

As most currently used probiotics for the treatment of RIOM were obtained from the GI tract, whether probiotics obtained from the oral cavity could exert superior radioprotective effects are not evidenced. Our group have shown that *S. salivarious* K12 as a representative oral probiotic strain can effectively reduce the incidence, delay the onset, shorten the duration, and alleviate the severity of RIOM (Peng et al., 2024). Future efforts to comprehensively compare *S. salivarious* K12 with other commonly used gut-derived probiotic strains such as *L. brevis* CD2 with respect to oral colonization capability, microecological modulation activity and clinical efficacy are still needed. Besides, as most of the studies administered probiotics *per oral (* Sharma et al., 2012; De Sanctis et al., 2019; Jiang et al., 2019; Xia et al., 2021; Mirza et al., 2022), which is expected to more directly/potently act on the GI tract instead of oral microenvironment in a relatively short period, whether topical use (e.g. mouthwash or lozenge) may guarantee a persistent oral colonization and thus lead to an improved clinical efficacy still needs future efforts. A current meta-analysis has reported that probiotic cocktail (mixed strains) is better than single strain in the management of oral mucositis (Feng et al., 2022), which will also be a promising direction for future research and development.

## 5 Summary

Accumulating evidence has shown the association between RIOM and oral microbial ecology, and application of probiotics has shown beneficial effects on this disease. Although the clinical outcomes of probiotics vary by specific strains, way of delivery and regimen of radiotherapy, they can effectively alleviate RIOM and improve patients’ quality of life likely via inhibiting the overgrowth of opportunistic pathogens, regulating host immune response and promoting mucosal repair, thus representing a promising adjunctive therapy for the better management of RIOM.
